# Pulseless Electrical Activity Arrest in End-Stage Heart Failure Complicated by Cocaine-Associated Cardiotoxicity and Cerebellar Infarction: A Post-resuscitation Diagnostic Pitfall

**DOI:** 10.7759/cureus.106465

**Published:** 2026-04-05

**Authors:** Cristina Suarez Chiriboga, Roxana Lazarescu

**Affiliations:** 1 Internal Medicine, Wyckoff Heights Medical Center, New York, USA

**Keywords:** cardiogenic shock, cerebellar infarction, cocaine-associated cardiomyopathy, heart failure with reduced ejection fraction, post-cardiac arrest syndrome, posterior circulation stroke, pulseless electrical activity arrest, substance-associated cardiotoxicity

## Abstract

Pulseless electrical activity (PEA) arrest in patients with end-stage heart failure with reduced ejection fraction (HFrEF) most commonly reflects profound circulatory collapse rather than a primary arrhythmogenic event. Although post-resuscitation encephalopathy often prompts early neurologic prognostication, myocardial dysfunction and refractory shock frequently dominate outcomes in this population.

We report the case of a 59-year-old woman with end-stage HFrEF, severe multivessel coronary artery disease, chronic lung disease, HIV infection, and active cocaine use who presented with acute hypoxic respiratory failure and decompensated heart failure and subsequently developed prolonged PEA arrest. Her post-arrest course was complicated by refractory cardiogenic shock, focal cerebellar infarction with delayed MRI due to hemodynamic instability and vasopressor dependence, and multisystem organ failure.

Taken together, these findings underscore an important post-resuscitation diagnostic pitfall: neurologic abnormalities may coexist; however, refractory hemodynamic collapse often predominates. An integrated framework incorporating shock physiology, substance-associated cardiotoxicity, and competing neurologic injury is essential for accurate clinical assessment and informed goals-of-care discussions.

## Introduction

Pulseless electrical activity (PEA) arrest commonly represents terminal circulatory collapse in patients with advanced structural heart disease. Contemporary American Heart Association (AHA) post-cardiac arrest guidelines emphasize that hypotension and shock following return of spontaneous circulation (ROSC) are major drivers of mortality and require immediate hemodynamic stabilization and cause-directed therapy [1]. This concept is reinforced by the post-cardiac arrest syndrome framework, which identifies myocardial dysfunction and systemic ischemia-reperfusion injury as central contributors to persistent shock independent of neurologic injury [2].

Patients with advanced heart failure with reduced ejection fraction (HFrEF) are particularly vulnerable to abrupt decompensation due to minimal physiologic reserve. The 2022 AHA/ACC/HFSA heart failure guideline characterizes advanced heart failure by recurrent hospitalizations, end-organ hypoperfusion, and high short-term mortality, especially following critical illness [3]. In this population, cardiac arrest most often reflects progressive pump failure rather than isolated electrical instability.

The management of cardiogenic shock requires structured escalation. The AHA scientific statement by van Diepen et al. outlines early recognition, vasopressor and inotropic support, and the consideration of mechanical circulatory support when appropriate while integrating goals-of-care discussions in patients with limited recovery potential [4]. European post-resuscitation guidance further stresses delaying definitive neurologic prognostication until confounding factors such as shock, sedation, and metabolic derangements are addressed [5]. Neuroprognostication guidelines similarly recommend the multimodal assessment and avoidance of premature conclusions in comatose post-arrest patients [6].

Cocaine exposure adds further complexity through ischemic, catecholaminergic, and arrhythmogenic mechanisms. Schwartz et al. describe cocaine-associated coronary vasoconstriction, increased myocardial oxygen demand, myocarditis, and cardiomyopathy [7]. Alawoè et al. review cocaine-related heart failure and therapeutic considerations [8], while Arenas et al. demonstrate an association between cocaine use and cardiomyopathy in a systematic review and meta-analysis [9]. Case literature additionally documents severe cocaine-associated cardiomyopathy presenting with refractory shock and heart failure [10].

Neurologic deterioration following cardiac arrest is frequently attributed to global hypoxic-ischemic brain injury; however, focal ischemic stroke may occur concurrently and represents an important diagnostic pitfall. Cronberg et al. review the heterogeneous spectrum of post-cardiac arrest brain injury extending beyond diffuse anoxia [11]. Wallin et al. demonstrated that acute focal brain lesions are commonly identified in survivors of cardiac arrest and may be clinically indistinguishable from global hypoxic-ischemic injury when early neuroimaging is not obtained [12]. Powers et al. emphasize timely neuroimaging in suspected stroke even in complex post-arrest settings [13].

Posterior circulation infarcts pose unique diagnostic challenges. Oppenheim et al. described false-negative diffusion-weighted MRI in acute ischemic stroke [14], and Edlow et al. further synthesized technical and timing limitations of posterior fossa imaging [15]. Finally, in patients with treated HIV, persistent immune activation and endothelial dysfunction may increase cerebrovascular risk despite virologic suppression, as reviewed by Hunt [16].

This case illustrates posterior circulation ischemia in the setting of refractory cardiogenic shock and end-stage HFrEF, with MRI acquisition delayed due to hemodynamic instability and ongoing vasopressor dependence.

## Case presentation

A 59-year-old woman with end-stage heart failure with reduced ejection fraction (EF: 10%) and ischemic cardiomyopathy with prior multivessel percutaneous coronary interventions, living with virologically controlled HIV (CD4 count, 136 cells/μL; reference range, 500-1,500 cells/μL) and with ongoing cocaine use, presented with acute hypoxic respiratory failure and decompensated heart failure.

On day 1, her oxygen saturation was 77% on room air, and she was tachypneic despite bronchodilators, corticosteroids, and diuretics. Noninvasive ventilation escalated from continuous positive airway pressure (CPAP) to bilevel positive airway pressure (BiPAP) without sufficient improvement. Within hours, she suffered a pulseless electrical activity (PEA) arrest. Advanced cardiac life support achieved return of spontaneous circulation (ROSC) after 16 minutes.

She was admitted to the ICU and supported with norepinephrine and milrinone for persistent cardiogenic shock. Key laboratory findings obtained during hospitalization are summarized in Table 1.

**Table 1:** Laboratory findings during hospitalization. Laboratory values obtained during hospitalization after pulseless electrical activity arrest with corresponding adult reference ranges. Abnormalities reflect myocardial injury, advanced heart failure, and metabolic disturbances related to cardiogenic shock. MB: myocardial band

Laboratory Test	Patient Result	Reference Range
B-type natriuretic peptide (BNP)	4818.2 pg/mL	<100 pg/mL
High-sensitivity troponin	40.3 ng/L	<14 ng/L
Creatine kinase (CK)	640 U/L	30-200 U/L
CK-MB	95.4 ng/mL	0-7.2 ng/mL
CK-MB index	14.90%	<5%
White blood cell count	6.5 × 10³/µL	4.0-11.0 × 10³/µL
Red blood cell count	3.56 × 10⁶/µL	4.0-5.2 × 10⁶/µL
Hemoglobin	8.5 g/dL	12-16 g/dL
Hematocrit	29.60%	36%-46%
Mean corpuscular volume (MCV)	83.1 fL	80-100 fL
Mean corpuscular hemoglobin (MCH)	23.9 pg	27-33 pg
Mean corpuscular hemoglobin concentration (MCHC)	28.7 g/dL	32-36 g/dL
Red cell distribution width (RDW)	18.90%	11.5%-14.5%
Platelet count	116 × 10³/µL	150-450 × 10³/µL
Sodium	141 mmol/L	135-145 mmol/L
Potassium	4.2 mmol/L	3.5-5.0 mmol/L
Chloride	108 mmol/L	98-107 mmol/L
Bicarbonate (CO₂)	28 mmol/L	22-29 mmol/L
Blood urea nitrogen	19 mg/dL	7-20 mg/dL
Creatinine	0.91 mg/dL	0.6-1.3 mg/dL
Glucose	96 mg/dL	70-100 mg/dL
Calcium	8.8 mg/dL	8.6-10.2 mg/dL
Magnesium	1.9 mg/dL	1.7-2.2 mg/dL
Albumin	2.6 g/dL	3.5-5.0 g/dL
Total protein	6.2 g/dL	6.4-8.3 g/dL
Aspartate aminotransferase (AST)	114 U/L	10-40 U/L
Alanine aminotransferase (ALT)	22 U/L	7-56 U/L
Arterial pH	7.43	7.35-7.45
Partial pressure of carbon dioxide (pCO₂)	52 mmHg	35-45 mmHg
Partial pressure of oxygen (pO₂)	118 mmHg	75-100 mmHg
Bicarbonate (HCO₃⁻)	34.5 mmol/L	22-26 mmol/L
Oxygen saturation	98.80%	>95%
Base excess	8.5	-2 to +2

On day 2, off sedation, she appeared lethargic, with minimally reactive pupils, preserved brainstem reflexes, and movements limited to withdrawal from pain. EEG demonstrated moderate generalized slowing without epileptiform activity.

**Figure 1:**
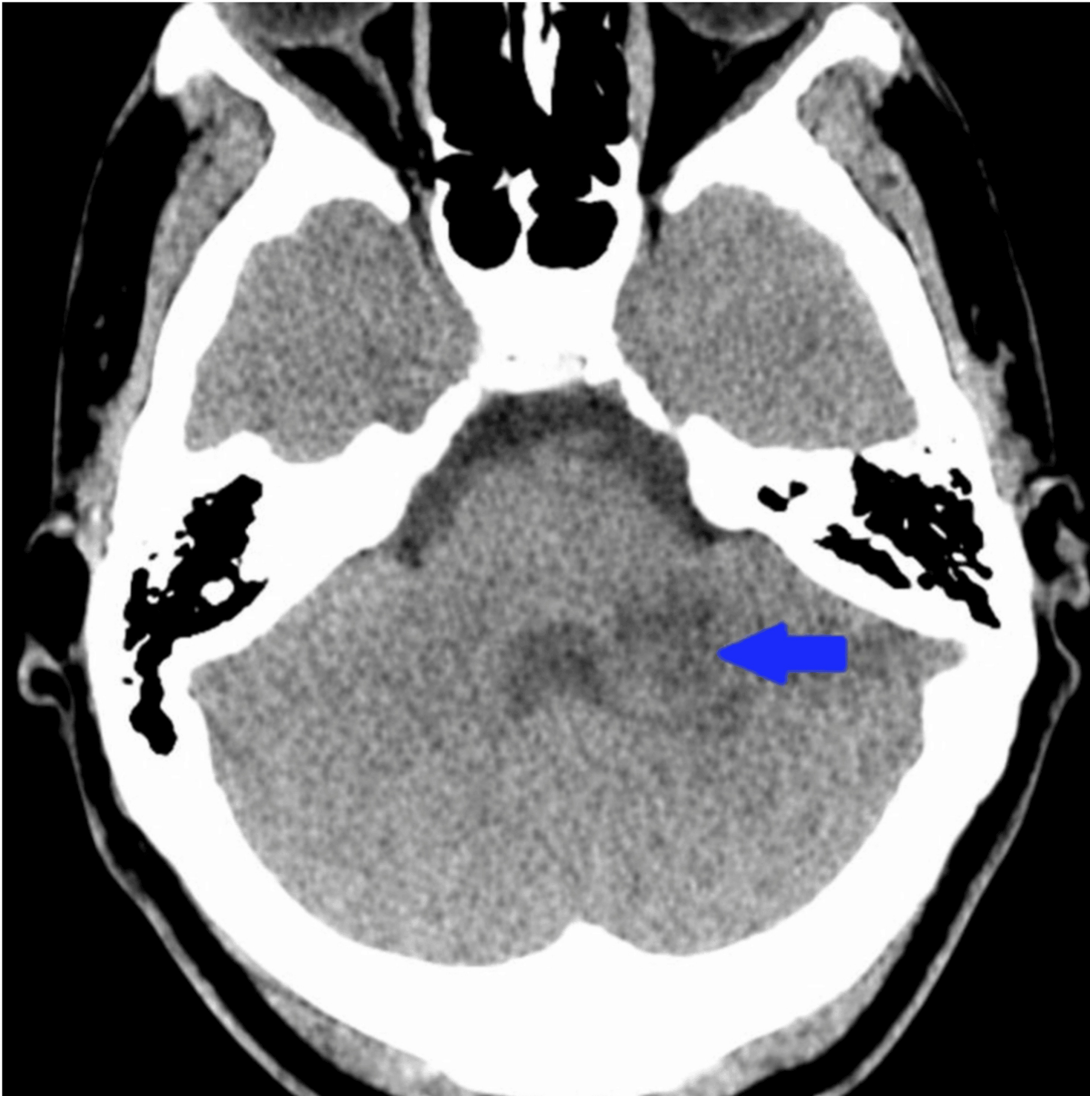
Noncontrast axial CT of the brain demonstrating left cerebellar infarction. Initial noncontrast head CT shows an approximately 3 cm hypodense lesion in the left cerebellar hemisphere, consistent with acute to subacute infarction, without hydrocephalus or significant mass effect.

Noncontrast CT of the head demonstrated a 3 cm hypodense lesion in the left cerebellar hemisphere, consistent with an acute-to-subacute infarct (Figure 1).

**Figure 2:**
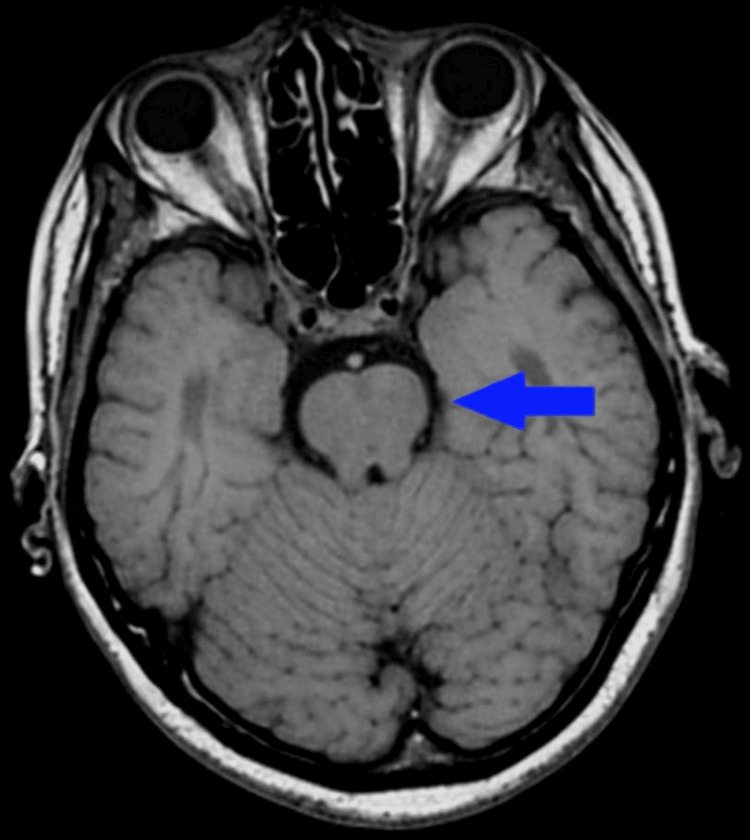
Noncontrast MRI of the brain four days after return of spontaneous circulation showing no acute diffusion restriction. MRI obtained approximately four days post-return of spontaneous circulation shows no acute diffusion restriction; imaging was delayed due to hemodynamic instability until clinical stabilization.

CT angiography showed patent posterior circulation without large-vessel obstruction. MRI performed on day 4, delayed due to hemodynamic instability, showed no acute diffusion restriction (Figure 2), highlighting the known limitations of delayed posterior circulation imaging.

**Figure 3:**
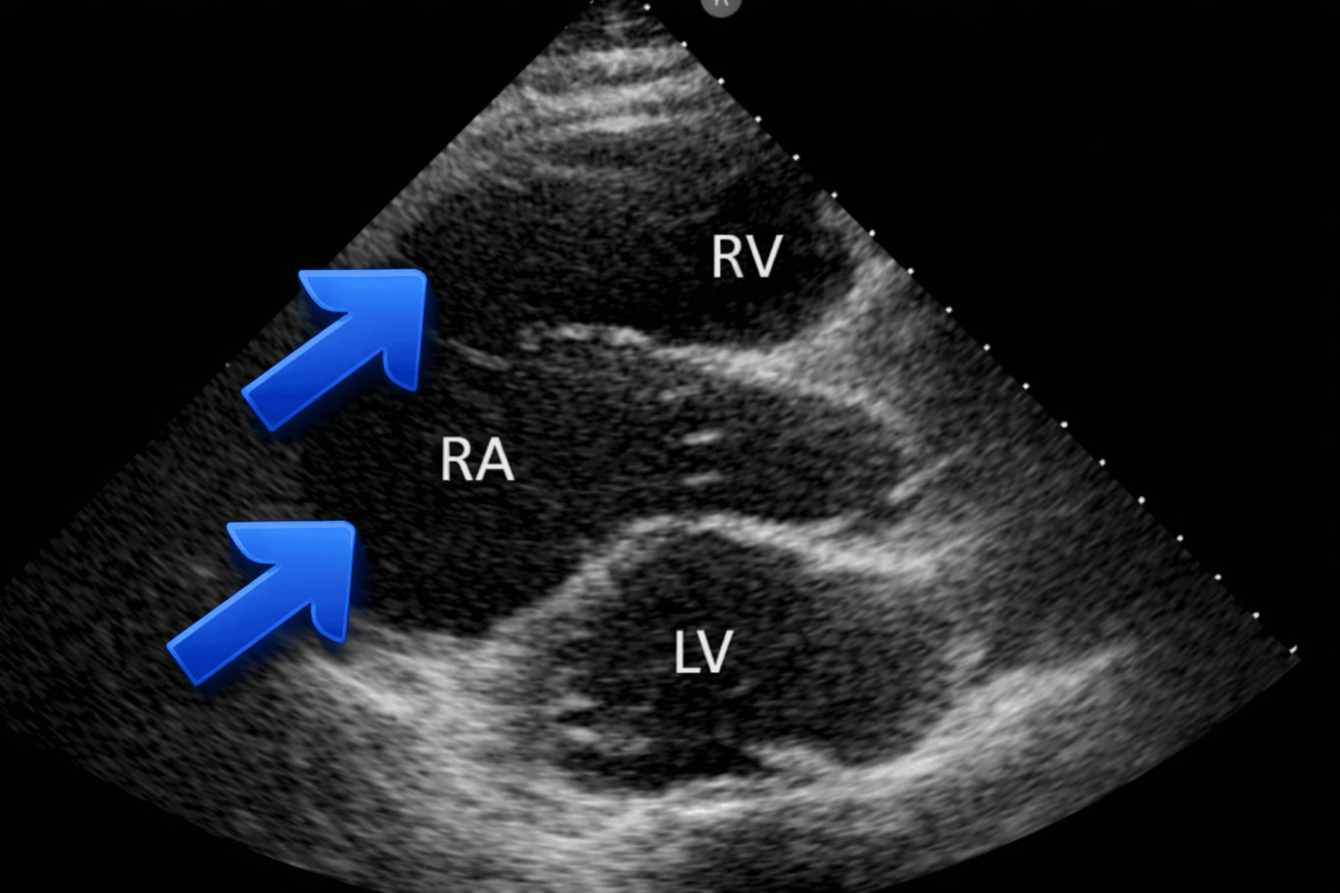
Transthoracic echocardiogram demonstrating advanced cardiomyopathy. Parasternal long-axis view shows marked right atrial (RA) and right ventricular (RV) dilation with severely reduced left ventricular (LV) systolic function, consistent with end-stage ischemic cardiomyopathy.

Transthoracic echocardiography demonstrated a marked dilation of the right atrium and right ventricle with severely reduced left ventricular systolic function (Figure 3). The left ventricle appeared globally hypokinetic with severe systolic dysfunction, and the estimated ejection fraction was approximately 10%, consistent with end-stage ischemic cardiomyopathy. These findings reflect advanced biventricular remodeling and severely impaired myocardial contractility, explaining the patient’s profound hemodynamic instability and cardiogenic shock requiring vasopressor and inotropic support.

**Figure 4:**
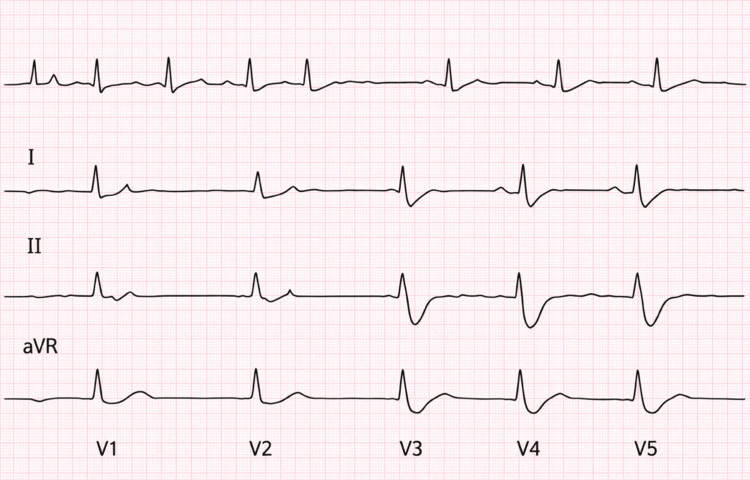
Electrocardiogram (ECG) demonstrating prior anterior myocardial infarction. ECG shows Q waves in V1-V4 with poor R-wave progression, consistent with prior anterior myocardial infarction and advanced ischemic cardiomyopathy. aVR: augmented vector right

Electrocardiography demonstrated Q waves in leads V1-V4 with poor R-wave progression (Figure 4), findings consistent with prior anterior myocardial infarction and chronic ischemic remodeling of the left ventricle. These electrocardiographic abnormalities correlate with the patient’s known history of multivessel coronary artery disease requiring prior percutaneous coronary interventions and support the diagnosis of advanced ischemic cardiomyopathy as the underlying substrate for her severe heart failure.

**Figure 5:**
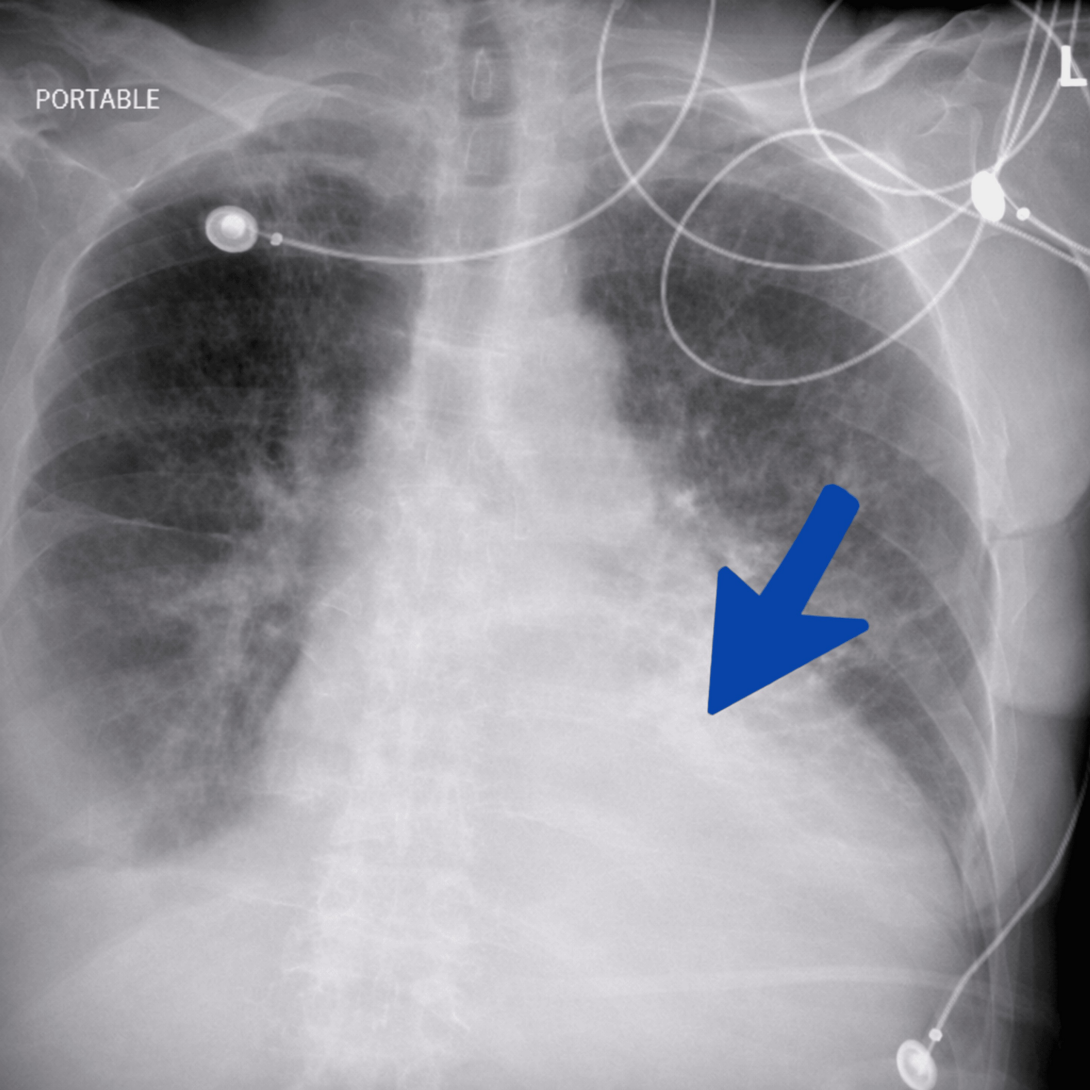
Portable chest radiograph demonstrating cardiomegaly and pulmonary congestion. A portable anteroposterior chest radiograph shows cardiomegaly with bilateral bibasilar opacities, consistent with pulmonary congestion in advanced heart failure.

Portable chest radiography demonstrated cardiomegaly with persistent bilateral bibasilar pleural-parenchymal opacities (Figure 5). These findings were consistent with pulmonary vascular congestion and fluid overload in the setting of acute decompensated heart failure. The enlarged cardiac silhouette reflects chronic structural remodeling associated with long-standing cardiomyopathy, while the bibasilar opacities likely represent a combination of pulmonary edema and dependent atelectasis frequently observed in critically ill patients with severe cardiac dysfunction.

Taken together, the imaging and electrocardiographic findings demonstrated advanced structural and functional cardiac disease consistent with end-stage ischemic cardiomyopathy complicated by refractory cardiogenic shock following pulseless electrical activity arrest.

## Discussion

This case highlights a potential post-resuscitation diagnostic pitfall in advanced heart failure: neurologic abnormalities may coexist after cardiac arrest, while refractory cardiogenic shock and myocardial dysfunction often contribute significantly to prognosis. The AHA identifies post-cardiac arrest shock as a major determinant of short-term survival and emphasizes prompt hemodynamic stabilization rather than an early attribution of outcomes solely to neurologic injury [1]. This is supported by the post-cardiac arrest syndrome framework, which describes myocardial dysfunction and ischemia-reperfusion injury as key contributors to persistent circulatory compromise [2]. The 2022 AHA/ACC/HFSA guideline similarly characterizes advanced HFrEF by limited physiologic reserve and reduced recovery potential following critical illness [3].

The management of cardiogenic shock includes vasoactive support with concurrent prognostic assessment and goals-of-care discussions [4,5]. Neuroprognostication guidelines recommend avoiding early neurologic conclusions in the presence of confounders such as shock and sedation [6].

Active cocaine use may have contributed through vasoconstriction, arrhythmogenesis, and myocardial injury [7]. Cocaine-associated heart failure is recognized as a multifactorial process involving ischemia, catecholamine toxicity, myocarditis, and cardiomyopathy [8,9], with prior reports describing severe presentations complicated by shock [10].

Cocaine use may also be considered within the broader context of social determinants of health, including socioeconomic instability, limited access to care, and chronic psychosocial stress, which are associated with adverse cardiovascular outcomes. This perspective may provide additional context for disease burden.

Although early CT demonstrated focal cerebellar infarction, MRI was delayed due to hemodynamic instability and vasopressor dependence; subsequent MRI showed no acute diffusion restriction. This is consistent with known limitations of delayed MRI in posterior circulation ischemia [14,15]. Neurologic impairment in this case was likely multifactorial, occurring in the setting of hypoxia, prolonged low-flow state during PEA arrest, metabolic derangements, sedation, and refractory cardiogenic shock, consistent with prior descriptions [11]. Persistent immune dysfunction in treated HIV may have also contributed to cerebrovascular vulnerability [16].

The early identification of focal infarction supported comprehensive neurologic evaluation and may help avoid premature prognostication, consistent with existing recommendations [6,12,13]. The present case is compared with prior reports of cocaine-associated cardiomyopathy and post-cardiac arrest presentations (Table 2).

**Table 2:** Comparison of the present case with representative published cocaine-associated cardiomyopathy and heart failure literature. The table summarizes clinical characteristics and outcomes, emphasizing the predominant role of refractory cardiogenic shock and limited cardiac reserve following pulseless electrical activity (PEA) arrest in cocaine-associated myocardial disease. M, male; F, female; HFrEF, heart failure with reduced ejection fraction; EF, ejection fraction

Clinical Characteristic	Present Case	Elkattawy et al. [10]	Arenas et al. [9]
Age/sex	59/F	38/M	Meta-analysis (multiple cohorts)
Cocaine exposure	Active and recurrent	Active	Chronic exposure
Cardiac pathology	End-stage ischemic HFrEF (EF: 10%)	Severe dilated cardiomyopathy	Increased risk of cardiomyopathy
Arrest/shock	Prolonged PEA with refractory cardiogenic shock	Cardiogenic shock	Elevated heart failure risk
Neurologic injury	Focal cerebellar infarction with delayed MRI	Not reported	Not reported
Outcome	Multisystem failure	Severe heart failure requiring ICU	Increased heart failure incidence

This case illustrates the interplay of refractory cardiogenic shock, cocaine-associated cardiotoxicity, and posterior circulation infarction, with delayed MRI contributing to diagnostic complexity in post-cardiac arrest neurologic assessment.

## Conclusions

Post-cardiac arrest encephalopathy should not be automatically attributed to global hypoxic-ischemic injury. Early CT imaging may identify focal ischemic lesions not apparent on MRI performed later in the clinical course, particularly in posterior circulation strokes when MRI acquisition is necessarily delayed due to ongoing hemodynamic instability and vasopressor dependence. In patients with end-stage HFrEF, refractory cardiogenic shock and limited cardiac reserve frequently remain the primary determinants of outcome. Maintaining a broad neurologic differential while prioritizing hemodynamic stabilization is essential to avoid premature prognostication and to support informed, patient-centered goals-of-care discussions.
